# Mmf1p Couples Amino Acid Metabolism to Mitochondrial DNA Maintenance in *Saccharomyces cerevisiae*

**DOI:** 10.1128/mBio.00084-18

**Published:** 2018-02-27

**Authors:** Dustin C. Ernst, Diana M. Downs

**Affiliations:** a Department of Microbiology, University of Georgia, Athens, Georgia, USA; National Cancer Institute

**Keywords:** 2-aminoacrylate, RidA, enamine deaminase, metabolite stress, mitochondrial genome

## Abstract

A variety of metabolic deficiencies and human diseases arise from the disruption of mitochondrial enzymes and/or loss of mitochondrial DNA. Mounting evidence shows that eukaryotes have conserved enzymes that prevent the accumulation of reactive metabolites that cause stress inside the mitochondrion. 2-Aminoacrylate is a reactive enamine generated by pyridoxal 5′-phosphate-dependent α,β-eliminases as an obligatory intermediate in the breakdown of serine. In prokaryotes, members of the broadly conserved RidA family (PF14588) prevent metabolic stress by deaminating 2-aminoacrylate to pyruvate. Here, we demonstrate that unmanaged 2-aminoacrylate accumulation in *Saccharomyces cerevisiae* mitochondria causes transient metabolic stress and the irreversible loss of mitochondrial DNA. The RidA family protein Mmf1p deaminates 2-aminoacrylate, preempting metabolic stress and loss of the mitochondrial genome. Disruption of the mitochondrial pyridoxal 5′-phosphate-dependent serine dehydratases (Ilv1p and Cha1p) prevents 2-aminoacrylate formation, avoiding stress in the absence of Mmf1p. Furthermore, chelation of iron in the growth medium improves maintenance of the mitochondrial genome in yeast challenged with 2-aminoacrylate, suggesting that 2-aminoacrylate-dependent loss of mitochondrial DNA is influenced by disruption of iron homeostasis. Taken together, the data indicate that Mmf1p indirectly contributes to mitochondrial DNA maintenance by preventing 2-aminoacrylate stress derived from mitochondrial amino acid metabolism.

## IMPORTANCE

Deleterious reactive metabolites are produced as a consequence of many intracellular biochemical transformations. Importantly, reactive metabolites that appear short-lived *in vitro* have the potential to persist within intracellular environments, leading to pervasive cell damage and diminished fitness. To overcome metabolite damage, organisms utilize enzymatic reactive-metabolite defense systems to rid the cell of deleterious metabolites. In this report, we describe the importance of the RidA/YER057c/UK114 enamine/imine deaminase family in preventing 2-aminoacrylate stress in yeast. *Saccharomyces cerevisiae* lacking the enamine/imine deaminase Mmf1p was shown to experience pleiotropic growth defects and fails to maintain its mitochondrial genome. Our results provide the first line of evidence that uncontrolled 2-aminoacrylate stress derived from mitochondrial serine metabolism can negatively impact mitochondrial DNA maintenance in eukaryotes.

## INTRODUCTION

RidA/YER057c/UK114 (Rid) family proteins (PF14588) are ubiquitous; phylogenetic analysis identified the archetypal RidA throughout all three domains of life, with additional subgroups (Rid1 to Rid7) present in prokaryotes (1, 2). Biochemical genetic studies in the bacterium *Salmonella enterica* determined that RidA proteins are deaminases that hydrolyze the reactive enamine 2-aminoacrylate (2AA), and other enamine/imine substrates, to ketoacids (3–8). In cellular metabolism, 2AA is generated by pyridoxal 5′-phosphate (PLP)-dependent α,β-eliminase enzymes as an intermediate in the conversion of amino acids to pyruvate (3, 4, 7). Following release from the enzyme, 2AA can be spontaneously converted to pyruvate by solvent water (7). Although 2AA can covalently modify enzymes *in vitro* (9–15), the potential for intracellular enamine damage was initially dismissed because of the short (~1.5-s) half-life of 2AA in water (16). However, biochemical and genetic data demonstrate that in the absence of RidA, unbound 2AA persists *in vivo* and inactivates PLP-dependent enzymes (1, 17, 18). These data indicate that the cellular milieu lacks sufficient free water to rapidly hydrolyze 2AA and provides the framework for phenotypes displayed by *ridA* mutants (6, 17–20). The prevalence of PLP-dependent enzymes (including α,β-eliminases) involved in basic metabolic pathways, coupled with the broad distribution of RidA homologues, suggests that 2AA stress likely occurs in diverse organisms.

Little is known about eukaryotic Rid proteins, although early reports suggested involvement in a variety of cellular processes mediated by undefined mechanisms (21–30). Interestingly, the mitochondrial RidA protein Mmf1p (mitochondrial matrix factor) influences mitochondrial DNA (mtDNA) stability in *Saccharomyces cerevisiae* (29, 30). Unlike some mitochondrial DNA maintenance factors (e.g., Abf2p) (31), Mmf1p does not bind and stabilize the mitochondrial nucleoid directly (29). Characterization of enamine deaminase activity *in vitro* and genetic complementation analyses *in vivo* suggest that RidA proteins from the three domains of life share a conserved cellular function (7, 28). The biochemical activity of RidA from yeast (e.g., Mmf1p) was not addressed by those previous studies. Here, we demonstrate that 2AA generation provokes the irreversible loss of mtDNA in *S. cerevisiae* lacking Mmf1p. The data indicate that iron present in the growth medium influences the stability of mtDNA when 2AA stress is encountered. Furthermore, 2AA stress elicits a growth defect that is distinct from the respiratory deficiency caused by mtDNA loss. This report establishes the role of Mmf1p in indirectly stabilizing mtDNA by preventing 2AA stress in a eukaryote and highlights damage that results from reactive metabolite imbalance in mitochondria.

## RESULTS

### Disruption of *MMF1* leads to a growth defect and loss of mtDNA.

**FIG 1:**
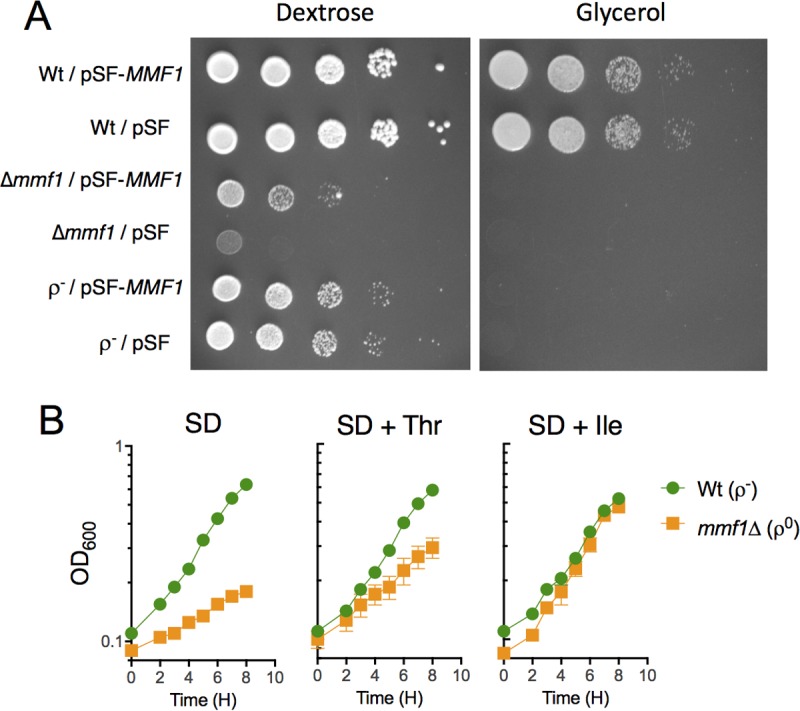
Yeast lacking Mmf1p cannot respire glycerol and have a growth defect on dextrose. (A) Growth of wild-type (ρ^+^), petite wild-type (ρ^−^), and *mmf1*Δ mutant strains on synthetic dextrose (SD) and synthetic glycerol (SG) solid media. An *MMF1* expression plasmid (pSF-*MMF1*) or the empty vector (pSF) was transformed into each strain prior to growth analyses. The numbers of cells from each strain were the same across the dilutions. (B) Growth of a ρ^0^ *mmf1*Δ mutant and petite wild-type (ρ^−^) strain in liquid SD medium supplemented with isoleucine or threonine as indicated. Data indicate averages and standard deviations of results from three independent cultures.

The *MMF1* locus of the haploid *S. cerevisiae* strain YJF153 (32) was replaced with a drug cassette by targeted gene disruption, and the drug marker was resolved to generate mutant strain DMy22 (*mmf1*Δ*0*). A plasmid expressing *MMF1* or an empty vector (pSF-TEF1-G418; Sigma) was introduced into DMy22, the wild-type parent (ρ^+^), and a chemically induced cytoplasmic petite (ρ^−^) strain derived from the wild-type strain. The resulting strains were assessed for growth on minimal medium containing a fermentable (dextrose [D]) or nonfermentable (glycerol [G]) carbon substrate (Fig. 1A). The *mmf1*Δ mutant had two significant growth phenotypes: (i) an inability to grow on glycerol and (ii) a reduced ability to grow on dextrose. Plasmid-borne *MMF1* failed to restore growth on glycerol, consistent with the irreversible loss or mutation of mtDNA observed in ρ^0^ or ρ^−^ cytoplasmic petites (33) (Fig. 1A). Results of deconvolution microscopy confirmed that the *mmf1*Δ mutant lacked detectable mtDNA and therefore was likely a ρ^0^-cytoplasmic petite (see below). In contrast, growth of the *mmf1*Δ mutant on dextrose was restored to a level similar to that of the ρ^−^ control by providing plasmid-borne *MMF1*. This indicated that transient metabolic deficiencies distinguishable from the respiratory defect were encountered by strains lacking Mmf1p. The addition of isoleucine and, to a lesser extent, threonine (an isoleucine precursor) restored growth of the ρ^0^ *mmf1*Δ mutant on dextrose (Fig. 1B) but not on glycerol. Together, these data distinguished the reversible and irreversible consequences of the *mmf1*Δ mutation, and each was considered in turn.

### Ilv1p-generated 2-aminoacrylate causes a growth defect in the absence of Mmf1p.

**FIG 2:**
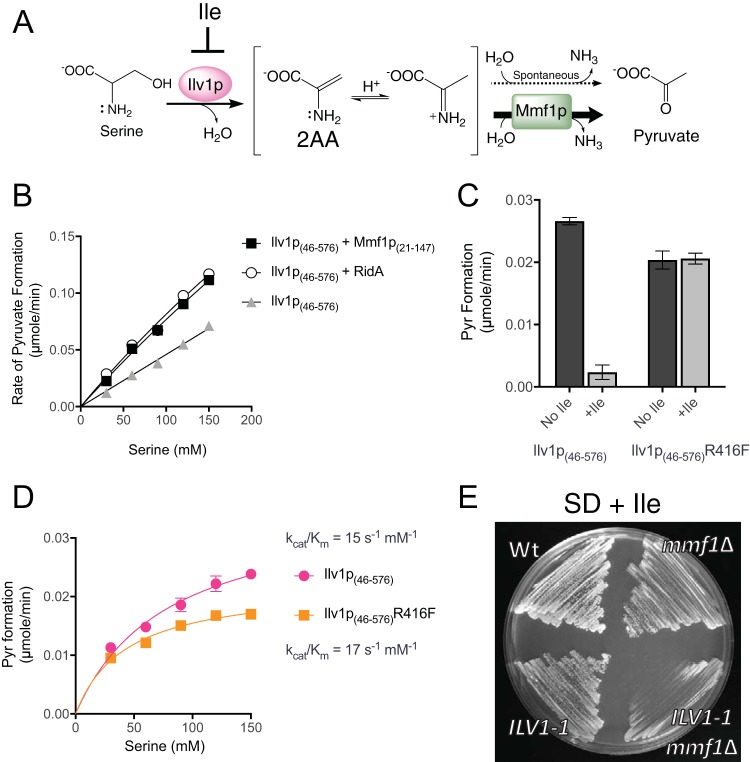
Ilv1p generates 2AA stress in yeast lacking Mmf1p. (A) Scheme of Ilv1p-mediated pyruvate formation, showing 2AA as an unbound intermediate that can be hydrolyzed to pyruvate by solvent water or Mmf1p. Isoleucine allosterically inhibits Ilv1p activity and prevents 2AA formation. Cha1p catalyzes the same reaction as Ilv1p but is not allosterically regulated. (B) The rates of conversion of serine to pyruvate by Ilv1p_(46−576)_ were enhanced similarly by adding Mmf1p_(21−147)_ (*S. cerevisiae*) or RidA (*S. enterica*). Data represent averages and standard deviations of results from three independent experiments, with error bars not exceeding the symbol boundaries. (C) The serine dehydratase activity of the purified Ilv1p_(46−576)_-R416F variant was insensitive to a concentration of isoleucine (3.3 mM) that completely inhibited the wild-type enzyme. Data represent averages and standard deviations of results from three independent experiments. (D) Ilv1p_(46−576)_-R416F has levels of catalytic efficiency for serine dehydration similar to those seen with the wild-type enzyme. Data indicate averages and standard deviations of results from three independent experiments. (E) Inserting the *ILV1-1* allele encoding Ilv1p^R416F^ into a ρ0 *mmf1*Δ strain prevented isoleucine from restoring full growth to the double mutant compared to the ρ° *mmf1*Δ single mutant following 48 h of incubation on solid medium consisting of SD plus Ile (SD + Ile) (1 mM) at 30°C. Wt, wild type.

The data support a model where both the growth defect and mtDNA loss are consequences of the toxic accumulation of 2AA in mitochondria lacking Mmf1p. The growth defect of a ρ^0^ *mmf1*Δ mutant on dextrose is reminiscent of a *ridA* mutant phenotype in *S. enterica* (19). In this case, 2AA generated by serine/threonine dehydratase (IlvA; EC 4.3.1.19) accumulates and compromises growth. Isoleucine allosterically inhibits IlvA, prevents 2AA generation, and restores growth in defined medium. Two nuclearly encoded serine/threonine dehydratases (EC 4.3.1.19) are active in the *S. cerevisiae* mitochondrion. Ilv1p is the biosynthetic serine/threonine dehydratase required for isoleucine biosynthesis (34), and Cha1p is a catabolic dehydratase induced by serine or threonine (35). Much like the bacterial enzyme IlvA, Ilv1p catalyzes the first committed step in isoleucine biosynthesis, is subject to feedback inhibition by isoleucine, and uses serine as a substrate as an alternative to threonine (36) (Fig. 2A). *In vitro*, Ilv1p dehydrated serine and released 2AA, which Mmf1p used as a substrate, leading to an increased rate of pyruvate formation (Fig. 2B). On a per-mole basis, the 2AA-hydrolyzing activity of Mmf1p was indistinguishable from that of the well-characterized RidA enzyme from *S. enterica* (Fig. 2B). These data support the hypothesis that the absence of Mmf1p leads to accumulation of 2AA following serine dehydration.

Taken together, the data favored the scenario depicted in Fig. 2A and suggested that the growth-stimulating role of isoleucine was exerted via the allosteric inhibition of Ilv1p. If true, preventing allosteric inhibition of Ilv1p would abolish the benefit of the presence of isoleucine in cells lacking Mmf1p. To test this hypothesis, an allosterically resistant variant of Ilv1p (Ilv1p^R416F^) was generated using an allosterically resistant variant of *Escherichia coli* IlvA (IlvA^R362F^) as a template (37). *In vitro*, recombinant Ilv1p_(46−576)_^R416F^ was insensitive to isoleucine concentrations that completely inhibited the wild-type enzyme (Fig. 2C). Importantly, the catalytic efficiency of serine dehydration by the variant enzyme was not significantly different from the efficiency seen with the wild-type enzyme (Fig. 2D). Wild-type *ILV1* was replaced with the full-length allele (*ILV1-1*) encoding Ilv1p^R416F^ to generate a strain where 2AA production by Ilv1p could not be inhibited. Isoleucine failed to completely reverse the growth defect of the *mmf1*Δ mutant strain expressing Ilv1p^R416F^ in minimal synthetic dextrose (SD) medium (Fig. 2E). Therefore, isoleucine improves growth of the *mmf1*Δ mutant strain in part through allosteric inhibition of Ilv1p and not by satisfying an isoleucine requirement. Taken together, these data show that Ilv1p generates 2AA from endogenous serine and that growth is limited unless Mmf1p or isoleucine quenches 2AA or inhibits Ilv1p activity, respectively.

### Cha1p increases 2-aminoacrylate stress when exogenous serine is present.

**FIG 3:**
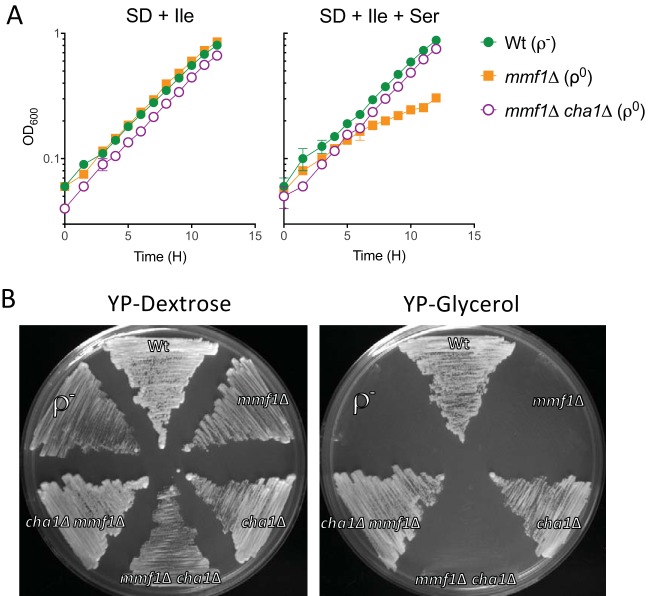
Cha1p contributes to 2AA generation when serine is provided exogenously. (A) Disruption of *CHA1* prevents conversion of exogenous serine (5 mM) to 2AA, thereby alleviating exogenous serine sensitivity in a ρ^0^ *mmf1*Δ *cha1*Δ background. Data are displayed as averages and standard deviations of results from three independent experiments. (B) Preventing 2AA production by simultaneously inhibiting the activity of Ilv1p (with isoleucine) and deleting *CHA1* renders Mmf1p nonessential for mtDNA maintenance despite the presence of serine in the growth medium. All mutants were constructed in the order indicated by the genotype and selected on YPD medium prior to streaking on the displayed YPD or YPG plates. The *mmf1Δ cha1Δ* strain (DMy17) was constructed independently from the *cha1Δ mmf1Δ* strain (DMy20), and the order in which the mutations were introduced differed as indicated in the text. Growth was recorded after 48 h at 30°C.

The ρ^0^ *mmf1*Δ mutant characterized as described above was constructed on rich (yeast extract-peptone-dextrose [YPD]) medium containing isoleucine, so feedback inhibition prevented Ilv1p from generating significant 2AA. Therefore, if 2AA stress were to contribute to mtDNA loss, an additional enzyme would be required to generate 2AA in the presence of isoleucine. The catabolic Ser/Thr dehydratase Cha1p is a logical source of 2AA, since *CHA1* expression is induced by serine, which is present in YPD, and since the enzyme is insensitive to regulation by isoleucine (35). Growth analysis in SD medium plus isoleucine, with or without serine, confirmed that Cha1p contributed to 2AA stress in the absence of Mmf1p (Fig. 3A). Both the ρ^0^ *mmf1*Δ and ρ^0^ *mmf1*Δ *cha1*Δ mutants grew in medium with isoleucine, but the addition of serine compromised growth only of the ρ^0^ *mmf1*Δ mutant, indicating *CHA1* is required for sensitivity to exogenous serine. These data indicate that 2AA is produced following induction of Cha1p by exogenous serine and that Ilv1p is inhibited by isoleucine via feedback inhibition. The increased level of 2AA compromised growth in the absence of *MMF1*. Unlike the results seen with a *ridA* mutant in *S. enterica* (38), the growth defect of the ρ^0^ *mmf1*Δ mutant was not reversed by the addition of common nutritional supplements (i.e., amino acids or vitamins). This result suggests that the cellular deficiencies underpinning the 2AA-dependent growth defect of *mmf1*Δ mutant strains in minimal medium are more complex than those caused by a single compromised enzyme.

### Disruption of serine dehydratase-dependent 2-aminoacrylate production preserves mtDNA in the absence of *MMF1*.

**FIG 4:**
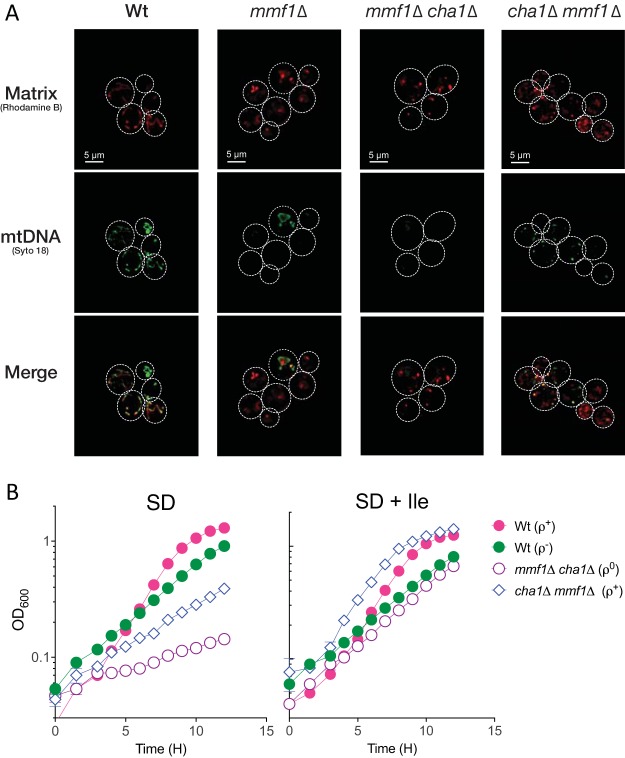
Mmf1p is dispensable for mtDNA maintenance in the absence of 2AA generators. (A) Microscopy (×100 magnification) confirms that the disruption of *MMF1* prior to *CHA1* led to loss of mtDNA following selection on YPD medium. Conversely, disruption of *CHA1* coupled with feedback inhibition of Ilv1p during propagation on YPD medium preserved mtDNA following the subsequent disruption of *MMF1*. (B) Although the ρ^+^ *cha1*Δ *mmf1*Δ mutant maintains respiratory capacity, it remains sensitive to moderate 2AA stress when Ilv1p activity is restored by removing isoleucine from the growth medium. Data displayed are averages and standard deviations of results from three independent cultures.

Despite the connection between Mmf1p and 2AA, it remained possible that the irreversible loss of mtDNA in an *mmf1*Δ mutant strain was unrelated to 2AA accumulation. However, data from order-dependent genetic manipulations and growth analyses demonstrate that 2AA stress specifically caused mtDNA loss (Fig. 3B). First, *CHA1* was disrupted in a ρ^+^ *MMF1* background. Second, *MMF1* was disrupted in the *cha1*Δ strain whereas Ilv1p was inhibited by isoleucine in the YPD-based selection medium. This resulted in a ρ^+^ *cha1*Δ *mmf1*Δ double mutant with unique properties. Significantly, introducing the genetic lesions in this order preempted the production of 2AA and resulted in a strain that respired glycerol (Fig. 3B) and maintained its mtDNA (Fig. 4A). Therefore, preventing both Ilv1p and Cha1p serine dehydratase activity renders Mmf1p nonessential for mtDNA maintenance. The inversely constructed *mmf1*Δ *CHA1* strain lost the ability to respire glycerol, and subsequent disruption of *CHA1* in the absence of *MMF1* did not restore growth on glycerol (Fig. 3B). These results demonstrate that disruption of *MMF1* prior to *CHA1* leads to permanent loss of mtDNA, a conclusion supported by mitochondrial staining of the ρ^0^ *mmf1Δ cha1Δ* strain (Fig. 4A). Thus, identical genotypes constructed in opposing series result in dramatically different outcomes with regard to mtDNA stability. These data support the conclusion that the preemptive disruption of *CHA1*, coupled with isoleucine-mediated inhibition of Ilv1p, blocks 2AA production in the mitochondrial matrix and bypasses the need for Mmf1p to maintain the mitochondrial genome.

The ρ^+^ *cha1*Δ *mmf1*Δ mutant maintained wild-type growth indefinitely when propagated on medium containing isoleucine. However, when the inhibition of Ilv1p was lifted by removing isoleucine, a growth defect of the ρ^+^ *cha1*Δ *mmf1*Δ mutant strain (µ = 0.18 ± 0.03) relative to the ρ^+^ wild-type control (µ = 0.45 ± 0.01) was detected (Fig. 4B). Isoleucine supplementation restored the growth rate (µ) of the ρ^+^ *cha1*Δ *mmf1*Δ strain (µ = 0.39 ± 0.04) to a level similar to that of the ρ^+^ wild-type control (µ = 0.48 ± 0.04). A parallel result was observed with the respiration-deficient ρ^0^ *mmf1*Δ *cha1*Δ mutant strain where the addition of isoleucine increased the growth rate to the level of the ρ^−^ wild-type control (µ = 0.26) (Fig. 4B). These data show that the ρ^+^ *cha1*Δ *mmf1*Δ mutant is susceptible to 2AA stress and experiences diminished growth when cultured on minimal medium. However, the respiratory capacity of the ρ^+^ *cha1*Δ *mmf1*Δ mutant was maintained indefinitely during growth on minimal medium (see Fig. S1 in the supplemental material). This result suggests that mtDNA is stable in spite of the growth defect given the moderate level of 2AA stress generated by Ilv1p on minimal medium. Enhancing flux through Ilv1p to increase 2AA stress severely diminished the ability of the ρ^+^ *cha1*Δ *mmf1*Δ mutant to respire glycerol after growth on minimal medium supplemented with 5 mM serine (Fig. S1). Taken together, these data indicate that in the absence of Mmf1p, Ilv1p acts on endogenous serine to generate moderate 2AA stress that elicits a minor and reversible growth defect akin to the bacterial paradigm (5). However, exogenous serine stimulates production (via Ilv1p and/or Cha1p) of sufficient 2AA to cause irreversible loss of the mitochondrial genome.

10.1128/mBio.00084-18.2 FIG S1  Serine diminishes the respiratory capacity of a ρ^+^ *cha1*Δ *mmf1*Δ strain grown in minimal medium. Dilution (10^5^ to 10^1^) plating of DMy20 (ρ^+^ *cha1*Δ *mmf1*Δ) precultured for up to 72 h in minimal SD medium with 5 mM serine reveals a diminished capacity to respire glycerol relative to the minimal SD medium control. Download FIG S1, EPS file, 12.9 MB. Copyright © 2018 Ernst and Downs. 2018 Ernst and Downs This content is distributed under the terms of the Creative Commons Attribution 4.0 International license.

### Availability of iron influences mtDNA stability in the absence of Mmf1p.

**FIG 5:**
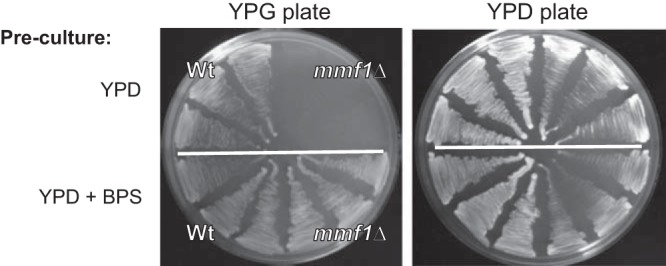
Chelation of iron in the preculture medium preserves the respiratory capacity of *mmf1*Δ mutants. A ρ^+^ *mmf1*Δ mutant strain was generated and propagated on YPG medium. Individual colonies from each preculture plate were streaked to YPD or YPD medium containing 10 µM BPS. After 48 h, three colonies were restreaked (alongside similarly precultured ρ^+^ wild-type cultures) on YPG or YPD medium to assess the impact of BPS on maintenance of respiratory capacity during growth on rich dextrose medium.

Given the precedents seen in bacterial systems, we theorized that the phenotypes observed for *mmf1*Δ mutants are caused by 2AA inactivation of PLP-dependent enzymes in the yeast mitochondrion. Several PLP-dependent enzymes in the yeast mitochondrion influence iron homeostasis (39, 40), and the disruption of iron homeostasis can lead to mitochondrial iron accumulation, enhanced oxidative stress, and diminished mtDNA stability (41, 42). Therefore, the impact of iron on 2AA-dependent loss of mtDNA was assessed. A ρ^+^ *mmf1*Δ (DMy41) mutant was made on yeast extract-peptone-glycerol (YPG) medium as described previously (29). Subsequent transfer of the ρ^+^ *mmf1*Δ mutant to YPD medium resulted in the loss of respiratory capacity (Fig. 5). However, transfer of the ρ^+^ *mmf1*Δ mutant to YPD medium containing the iron chelator bathophenanthrolinedisulfonic acid (BPS) preserved respiratory capacity (Fig. 5). These data indicate that the loss of mtDNA caused by 2AA stress is contingent on the presence of iron in the growth medium. Sequestration of iron in the growth medium using BPS prevented 2AA-dependent loss of mtDNA, perhaps reflecting that 2AA stress induces accumulation of iron in the mitochondria, leading to damage and eventual loss of mtDNA. These data offer a starting point for future characterization of targets modified by 2AA in the yeast mitochondrion.

## DISCUSSION

**FIG 6:**
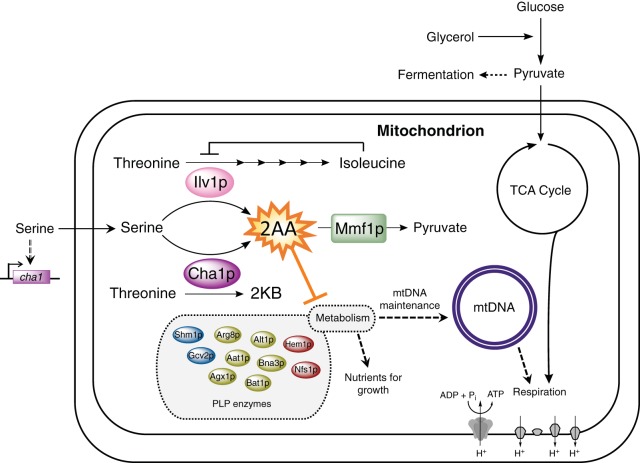
Model of 2AA stress in the yeast mitochondrion. PLP-dependent serine dehydratases active in the yeast mitochondrion (Ilv1p and Cha1p) generate 2AA through the dehydration of serine. Unless Mmf1p is present to prevent 2AA accumulation, metabolic stress arises, ultimately causing loss of the mitochondrial genome. The precedent for 2AA damaging PLP-dependent enzymes suggests that the negative influence of 2AA on mtDNA maintenance is indirect and likely due to damage of one or more target PLP-dependent enzymes. 2KB, 2-ketobutyrate; 2AA, 2-aminoacrylate; CHA1 and ILV1 encode serine/threonine dehydratases (EC 4.3.1.19).

Our work shows that accumulation of 2AA causes metabolic stress and loss of mtDNA in *S. cerevisiae*. Furthermore, Mmf1p prevents 2AA accumulation in the *S. cerevisiae* mitochondrion. Two consequences of 2AA accumulation in the mitochondrion were identified: the irreversible loss of mtDNA giving rise to respiration-deficient ρ^0^ cytoplasmic petites and a transient growth defect on fermentable carbon substrates. The latter phenotype is reminiscent of the growth defects caused by 2AA stress in *S. enterica* (6, 19) and other organisms (8, 28). PLP-dependent enzymes are the only targets of 2AA damage characterized to date (43), suggesting that the *mmf1*Δ strain growth defect is due to inhibition of one or more (of 10 possible) target PLP enzymes in the mitochondrion (44) (Fig. 6). 2AA tested with a bacterial system is not mutagenic *in vivo* (see Table S2 in the supplemental material), making it unlikely that direct DNA damage by 2AA causes the loss of mtDNA. We suggest that loss of mtDNA is caused by the stress that 2AA exerts on the mitochondrial metabolic network. Specifically, we favor a model in which 2AA damages multiple mitochondrial PLP-dependent enzymes, ultimately leading to destabilization and loss of the mitochondrial genome (Fig. 6). The independent disruption of mitochondrial PLP-dependent enzymes involved in heme biosynthesis (Hem1p; EC 2.3.1.37), iron-sulfur cluster biogenesis (Nfs1p; EC 2.8.1.7), one-carbon metabolism (Shm1p; EC 2.1.2.1), and aspartate metabolism (Aat1p; EC 2.6.1.1) has been reported to influence mtDNA stability to various degrees (40, 45–47). Notably, Hem1p and Nfs1p are directly involved in iron metabolism; damage to these enzymes caused by 2AA may underlie the iron sensitivity of *mmf1*Δ mutants. The iron sensitivity of the *mmf1*Δ mutant is reminiscent of that of yeast lacking the frataxin homologue *YFH1* (41, 42), consistent with mitochondrial iron accumulation giving rise to oxidative stress that damages mtDNA. Given the variety of PLP-dependent enzymes that individually impact iron homeostasis and mtDNA stability, it is reasonable to conjecture that the presence of a combination of partially defective enzymes could result in mtDNA loss. Further understanding of how 2AA leads to loss of mtDNA may uncover a novel stress response pathway in mitochondria.

Our work identified a lethal consequence of uncontrolled reactive metabolite accumulation in the mitochondrion. Synthesis of 2AA is unavoidable in PLP-dependent serine dehydration, resulting in the need for RidA proteins (e.g., Mmf1p) to prevent accumulation of this reactive metabolite. Strikingly, when *S. cerevisiae* is exposed to serine, *CHA1* and *MMF1* are the two most highly expressed genes (48), suggesting that these enzymes act in concert to safely reduce serine levels in the mitochondrion. Interestingly, Cha1p is reported to be a component of the mitochondrial nucleoid in *S. cerevisiae* (49), perhaps reflecting an added benefit of Cha1p-dependent physical stabilization of the nucleoid during periods of elevated serine catabolism and 2AA production. The Mmf1p homologue in humans, UK114 (PF01042), is variably described as a tumor antigen, calpain activator, or translation inhibitor in diverse animal cell types (21, 26, 50–52). Importantly, UK114 can substitute for Mmf1p to maintain the mitochondrial genome in *S. cerevisiae* (29), which argues in favor of an evolutionarily conserved biological function. PLP-dependent serine dehydratases are broadly distributed among eukaryotes, which emphasizes the breadth of 2AA stress (53–55). Furthermore, many cell types, including cancer cells and neurons, require high serine levels to promote growth and proliferation (56–58), predisposing certain cell types to high concentrations of a known 2AA precursor. Our work provides a framework for understanding the physiological role of Mmf1p and other eukaryotic RidA proteins, in addition to dissecting the mechanism by which 2AA stress causes loss of mtDNA.

## MATERIALS AND METHODS

### Strains, media, and chemicals.

*Saccharomyces cerevisiae* strain YJF153 (*MAT***a** HO::*dsdAMX4*) was derived from an oak tree isolate (YPS163) and provided by Justin Fay (Washington University) (32). Rich medium (YP) consisted of 20 g/liter peptone (Fisher Scientific) and 10 g/liter yeast extract (Becton Dickinson). Minimal medium (S medium) contained 1.7 g/liter yeast nitrogen base without amino acids or nitrogen (Sunrise Science; catalog no. 1500-100) and 5 g/liter ammonium sulfate. Either dextrose (D; 20 g/liter) or glycerol (G; 30 g/liter) was provided as the sole carbon source. Solid medium was made by adding 20 g/liter Difco agar (Becton Dickinson). Antibiotics used for deletion marker selection were added at the following final concentrations: 400 µg/ml Geneticin (G418; Gold Biotechnology), 200 µg/ml hygromycin B (Gold Biotechnology), and 100 µg/ml nourseothricin sulfate (cloNAT; Gold Biotechnology). A lower concentration of Geneticin (200 µg/ml) was used for maintenance of strains with confirmed G418 resistance. Isoleucine or threonine was added to minimal growth medium at a final concentration of 1 mM.

*Escherichia coli* strain BL21-AI was used for recombinant protein overproduction. Standard *E. coli* growth medium (LB broth) consisted of 10 g/liter tryptone, 5 g/liter yeast extract, and 10 g/liter NaCl. Superbroth containing tryptone (32 g/liter), yeast extract (20 g/liter), sodium chloride (5 g/liter), and sodium hydroxide (0.2 g/liter) was used when high cell densities were required for protein overproduction. Ampicillin (150 µg/ml) was added to the growth medium as needed. Reagents and chemicals were purchased from Sigma-Aldrich unless otherwise specified.

### Genetic techniques and growth methods.

Gene disruptions in *S. cerevisiae* were made following the standard gene replacement method described by Hegemann and Heick (59), resulting in the strains listed in Table 1 in the supplemental material. Disruption cassettes were amplified using the appropriate primers and plasmid templates listed in Table S1. Purified DNA (1 µg) was transformed into *S. cerevisiae* by incubating cells suspended in a mixture of 33% polyethylene glycol 3350 (PEG 3350), 100 mM lithium acetate, and 0.28 mg/ml salmon sperm DNA at 30°C for 30 min followed by 30 min of heat shock at 42°C. The transformed cells were recovered in rich medium containing dextrose (YPD) for 1 h at 30°C and were subsequently plated on solid YPD containing the relevant selection agent. Colonies that arose after 2 to 3 days of incubation were transferred to selective medium, and individual colonies were screened via PCR for the appropriate genetic recombinants. (Additional details of the methods used in the study are provided in Text 1 in the supplemental material.)

10.1128/mBio.00084-18.1 TEXT S1  Supplemental methods. Download TEXT S1, DOCX file, 0.1 MB. Copyright © 2018 Ernst and Downs. 2018 Ernst and Downs This content is distributed under the terms of the Creative Commons Attribution 4.0 International license.

10.1128/mBio.00084-18.3 TABLE S1  Plasmids and primers used in this study. Plasmids and primers used in the construction of strains for this study are displayed. Download TABLE S1, DOCX file, 0.3 MB. Copyright © 2018 Ernst and Downs. 2018 Ernst and Downs This content is distributed under the terms of the Creative Commons Attribution 4.0 International license.

**TABLE 1:** Strain list

Strain	Relevant genotype
YJF153^a^	*MAT***a** HO::*dsdAMX4*
DMy13	*mmf1*::*kanMX* (ρ^0^)
DMy16	*cha1*::*hphMX*
DMy17	*mmf1*::*kanMX cha1*::*hphMX* (ρ^0^)
DMy18	*ilv1*::*natMX*
DMy20	*cha1*::*hphMX mmf1*::*kanMX*
DMy23	YJF153 (ρ^−^)
DMy21	*mmf1*::*hphMX*-*loxP* (ρ^0^)
DMy22	*mmf1*Δ*0* (ρ^0^)
DMy31	YJF153/pDM1481
DMy32	YJF153/pSF-empty
DMy33	*mmf1*Δ0 (ρ^0^)/pDM1481
DMy34	*mmf1*Δ0 (ρ^0^)/pSF-empty
DMy35	YJF153 (ρ^−^)/pDM1481
DMy36	YJF153 (ρ^−^)/pSF-empty
DMy41	*mmf1*::*loxP-kanMX-loxP* (ρ^+^)
DMy43	*ilv1*Δ*0*::*ILV1*-1^b^
DMy46	*ilv1*Δ*0*::*ILV1*-1 *mmf1*::*kanMX*
DM15531	*E. coli* BL21-AI/pDM1463
DM15533	*E. coli* BL21-AI/pDM1467
DM15910	*E. coli* BL21-AI/pDM1536

^a^ All yeast strains were constructed in a YJF153 strain background.

^b^ Allele *ILV1-1* encodes an Ilv1p^R416F^ feedback-resistant variant.

For growth analyses, yeast strains were revived from −80°C freezer stocks and streaked for isolation on YPD. Single colonies were inoculated into 2-ml YPD cultures and incubated at 30°C with shaking (200 rpm) overnight. Turbid cultures were (i) 10-fold serially diluted with NaCl for spot plating (10 µl) on solid medium or (ii) inoculated (1%) into 5 ml of SD medium to monitor growth over time on the basis of the change in optical density at 600 nm (OD_600_). Growth curves were plotted as averages and standard deviations of results from three independent cultures using GraphPad Prism 7.0. Specific growth rates (µ) were calculated on the basis of the equation ln(*X*/*X*_0_)/*T*, where *X* represents OD_600_, *X*_0_ is the initial OD_600_ of the linear growth period monitored, and *T* is time in hours.

### Molecular techniques.

Plasmids were constructed using standard molecular techniques. DNA was amplified using Q5 DNA polymerase (New England Biolabs) with primers purchased from Eton Bioscience Inc. (Research Triangle Park, NC). Plasmids were isolated using a Wizard Plus SV miniprep kit (Promega), and PCR products were purified using an E.Z.N.A. DNA isolation kit (Omega BioTek). Restriction endonucleases used for molecular cloning were purchased from New England Biolabs. T4 ligase (Thermo Scientific) was used to ligate inserts to vectors. The plasmids and primers used are listed in Table S1. Plasmids pUG6, pUG74, and pUG75 were used as templates for drug cassette amplification (EUROSCARF). Plasmid pFA6a-*kanMX* was provided by David Garfinkel (University of Georgia). pSF episomal shuttle vector (Sigma-Aldrich; catalog no. OGS542) containing a *TEF1* promoter, *TPI1* terminator, and Geneticin (yeast)/ampicillin (bacteria) resistance markers was used to express *MMF1* in *S. cerevisiae* (pDM1481); full-length *MMF1* was PCR amplified for cloning using mmf1_pSF_NcoI_F and mmf1_pSF_XbaI_R. Constructs for protein overproduction were made using pET20b (Novagen) as the vector backbone. Primers were designed to amplify *MMF1* (mmf1_NdeI_F_truncated_pET20, mmf1_XhoI_R) and *ILV1* (ilv1_NdeI_F_truncated_pET20, ilv1_Not1_R_pET20) lacking the N-terminal mitochondrial targeting sequences and ligated into pET20b following restriction enzyme digestion of the insertion and vector, forming pDM1463 and pDM1467, respectively. The full-length allele of *ILV1* was cloned into pET20b to make pDM1469. All constructs were transformed into DH5α following ligation and selected on LB medium containing the appropriate drug. Plasmid insertions were confirmed by sequence analysis performed at Eton Bioscience Inc. Constructs pDM1467 and pDM1469 were used as templates for site-directed mutagenesis to generate pDM1536 and pDM1540, respectively. Site-directed mutants of *ILV1* were made by amplifying pDM1467 and pDM1469 with Q5 polymerase and primer ilv1_R416F, changing the codon for arginine-416 (AGA) to phenylalanine-416 (TTC), generating allele *ILV1-1*. Following transformation into DH5α, site-directed mutants were confirmed by Sanger sequencing (Eton Bioscience). Plasmid pDM1540 served as a template to amplify the *ILV1-1* allele for integration into the chromosome at the *ilv1*::*natMX* locus of DMy18. Replacement of the *natMX* drug cassette in DMy18 with the feedback-resistant *ILV1-1* allele restored isoleucine prototrophy to DMy43 and abolished nourseothricin sulfate resistance. Integration of the appropriate allele in DMy43 was confirmed by sequence analysis (Eton Bioscience Inc.).

### Purification of Mmf1p_(21−147)_.

Mmf1p_21-147_, lacking the N-terminal amino acids required for mitochondrial localization, was purified from an *E. coli* BL21-AI strain containing pDM1463. An overnight culture of DM15531 grown in 10 ml of superbroth containing ampicillin was inoculated into 2 liters of superbroth with ampicillin. Cultures were grown for 3 h at 37°C with aeration (200 rpm) until an OD_650_ of 0.5 was reached. Fresh arabinose was added to reach a final concentration of 0.02%, and cultures were shifted to 30°C and incubated for an additional 16 h while being shaken. Cells were harvested by centrifugation and resuspended in binding buffer consisting of 50 mM Tris-HCl (pH 8), 200 mM sodium chloride, 10 mM imidazole, 1 mM TCEP [Tris(2-carboxyethyl)phosphine], and 10% glycerol. Lysozyme (1 mg/ml), phenylmethylsulfonyl fluoride (100 g/ml), and DNase (25 g/ml) were added to the cell suspension, and the reaction mixture was incubated on ice for 1 h. Cells were lysed using a Constant Systems Limited One Shot (United Kingdom) system by passing cells through the disrupter one time with the pressure set to 21,000 lb/in^2^. Following lysis, the extract was clarified, filtered, and injected into a HisTrap high-performance (HP) Ni-Sepharose column (5 ml). The column was washed with five column volumes of binding buffer with 40 mM imidazole added. Mmf1p was eluted by increasing the concentration of imidazole from 40 to 300 mM over 10 column volumes, and 3-ml fractions were collected and analyzed by SDS-PAGE to determine protein purity. Fractions containing pure (>99%) protein were pooled and concentrated by centrifugation with a 4,000-molecular-weight-cutoff filter unit (Millipore). The concentrated protein sample was transferred to storage buffer containing 10 mM HEPES and 10% glycerol using a PD-10 desalting column (GE Healthcare). Protein yield as determined using the bicinchoninic acid (BCA) assay (Pierce) was approximately 14 mg/ml. Protein aliquots were frozen in liquid nitrogen and stored at −80°C.

### Purification of Ilv1p_(46-576)_ and Ilv1p_(46-576)_R416F.

Plasmids encoding His-tagged versions of Ilv1p_46-576_ (pDM1467) and Ilv1p_(46−576)_R416F (pDM1536) were transformed into *E. coli* BL21-AI for protein purification. The resulting strains were inoculated into 10 ml of superbroth containing ampicillin and grown overnight at 37°C. Overnight cultures were subcultured into 2 liters of superbroth containing ampicillin and grown at 37°C until an OD_650_ of 0.7 was reached. Arabinose (0.02%) was added to induce expression, and cultures were shifted to 30°C for 16 h. Cells were harvested at 4°C by centrifugation (15 min at 8,000 × *g*) and resuspended in binding buffer containing 50 mM potassium phosphate (pH 8), 500 mM sodium chloride, 10 mM imidazole, 1 mM TCEP, 10 µM PLP, and 10% glycerol. Lysozyme (1 mg/ml), phenylmethylsulfonyl fluoride (100 g/ml), and DNase (25 g/ml) were added to each cell suspension, which then sat on ice for 1 h. Cells were mechanically lysed using a French pressure cell (5 passes at 10,342 kPa). Each resulting lysate was clarified by centrifugation (45 min at 48,000 × *g*) and filtered through a membrane with 0.45-µm pores. Filtered lysates were loaded onto HisTrap HP Ni-Sepharose columns (5 ml), and the columns were washed with five column volumes of binding buffer containing 40 mM imidazole. Protein was eluted by increasing the concentration of imidazole in the elution buffer from 40 to 300 mM over 10 column volumes. Purified protein was concentrated by centrifugation with a 10,000-molecular-weight-cutoff filter unit (Millipore), and the buffer was replaced with 50 mM Tris-HCl (pH 7.5) containing 10 µM PLP and 10% glycerol using a PD-10 desalting column (GE Healthcare). Protein recovery as determined by the BCA assay (Pierce) was approximately 10.8 mg/ml for Ilv1p_46-576_ and 13 mg/ml for Ilv1p_(46−576)_R416F. Protein aliquots were frozen in liquid nitrogen and stored at −80°C.

### Ilv1p generation of pyruvate assays.

Ilv1p_(46−576)_ serine dehydratase activity was assayed in the presence or absence of Mmf1p or RidA from *S. enterica* as previously described (7). The activity of purified RidA was previously confirmed (3). Reaction mixtures (300 µl) consisted of 50 mM *N*-cyclohexyl-2-aminoethanesulfonic acid (CHES) (pH 9.5), 0.6 µM Ilv1p_(46−576)_, and Mmf1p_(21−147)_ (1.3 µM) or RidA (1.3 µM). Experiments were performed in triplicate, and the results were measured using a 96-well quartz plate and a SpectraMax M2 (Molecular Devices) microplate reader. Reactions were initiated by adding l-serine and monitored continuously at 230 nm for 120 s. Initial rates were determined on the basis of the increase in *A*_230_ corresponding to pyruvate production. A standard curve of pyruvate concentrations relative to *A*_230_ was generated and used to calculate reaction rates, reported as micromoles of pyruvate produced per minute. The data, reported as averages and standard deviations of results from three independent experiments, were fitted with curves on the basis of the Michaelis-Menten equation using GraphPad Prism 7.0. The procedure described above was used to compare the levels of Ilv1p_(46−576)_ and Ilv1p_(46−576)_R416F catalytic efficiency, with the sole change being that 50 mM potassium phosphate (pH 8) was used instead of 50 mM CHES (pH 9.5).

### Inhibition of Ilv1p variants by isoleucine assays.

The sensitivity of Ilv1p_(46−576)_ and Ilv1p_(46−576)_R416F to allosteric regulation by isoleucine was determined *in vitro*. Reaction mixtures (300 µl) consisted of 50 mM potassium phosphate (pH 8) and 0.6 µM Ilv1p_(46−576)_ or 0.6 µM Ilv1p_(46−576)_R416F. Isoleucine was added to assays at a final concentration of 3.3 mM. Experiments were performed in triplicate, and the results were measured using a 96-well quartz plate and a SpectraMax M2 (Molecular Devices) microplate reader. Reactions were initiated by adding 120 mM l-serine and monitored continuously at 230 nm for 120 s. Initial rates were determined on the basis of the increase in *A*_230_ corresponding to pyruvate formation. A standard curve of pyruvate concentrations relative to *A*_230_ was generated and used to calculate reaction rates, reported as micromoles of pyruvate produced per minute. The reaction rates for a single concentration of serine added are reported as averages and standard deviations of results from three independent experiments.

### Microscopy.

Strains were grown in YPD to full density overnight and then diluted to an OD_600_ of 0.1 in complete synthetic dextrose medium (Sunrise Science; catalog no. 1001-010) the following morning. Cultures were then grown at 30°C and 200 rpm for 4 h. Cells were harvested by centrifugation (2 min at 2,000 × *g*) and resuspended in mounting medium consisting of 10 mM HEPES (pH 7.4) and 5% dextrose. Cells were again pelleted and washed in an equal volume of mounting medium. The mitochondrial matrix was stained with 1 µM rhodamine B (Molecular Probes) for 20 min, followed by 3 min of staining with 10 µM SYTO 18 (Molecular Probes) to detect mitochondrial DNA. Cells were pelleted and resuspended in fresh mounting medium, to which 2 µl of ProLong Live Antifade reagent (Thermo Fisher) was added. Tubes containing cells and antifade reagent were transferred to a sealed box at 4°C overnight. The next morning, 20 µl of cell suspension was mounted on a glass slide and imaged using a DeltaVision microscope system (GE Life Sciences) equipped with an Olympus IX-71 inverted microscope and a xenon arc lamp as the illumination source for exciting the fluorophores. Rhodamine B was visualized with excitation at 555 nm and emission at 627 nm, and SYTO 18 was visualized with excitation at 490 nm and emission at 507 nm. Figures were generated by merging Z stacks of rhodamine B and SYTO 18 images, and the resulting merged images were deconvoluted using a conservative ratio and 15 processing cycles and automated softWoRx 6.5.2 (GE) image acquisition software. A quick projection was generated, and the resulting images were exported to Adobe Illustrator 21.0.2. Images were cropped and resized without adjusting features related to contrast or brightness. Cell borders were generated in Adobe Illustrator by outlining each cell boundary as determined from a white light snapshot.

### Impact of iron on respiratory capacity.

The *MMF1* locus was disrupted by transforming a ρ^+^ wild-type strain with a drug resistance cassette (*loxP-kanMx-loxP*), and recombinants were selected on YPG medium containing 280 µg/ml Geneticin. Subsequent propagation of the ρ^+^ *mmf1*Δ (DMy41) mutant was done on YPG medium to preserve the mitochondrial genome. The ρ^+^ *mmf1*Δ mutant and a wild-type control were revived from freezer stocks on solid YPG medium and grown overnight at 30°C. Single colonies were picked and streaked onto solid YPD medium or solid YPD medium containing 10 µM bathophenanthrolinedisulfonic acid (BPS). Cultures were incubated at 30°C for 48 h, and then three representative colonies were picked from each preculture and restreaked to YPG and YPD plates to assess respiratory capacity. Plates were incubated 48 h at 30°C, and respiration proficiency was assessed on the basis of the ability of strains to grow on glycerol (YPG)-containing medium.

10.1128/mBio.00084-18.4 TABLE S2  Frequency of rifampin-resistant colonies in wild-type and *ridA* strains of *S. enterica*. The values reported indicate the averages and standard deviations of results from three independent experiments. Download TABLE S2, DOCX file, 0.03 MB. Copyright © 2018 Ernst and Downs. 2018 Ernst and Downs This content is distributed under the terms of the Creative Commons Attribution 4.0 International license.
