# Supplementary material for: Mmf1p Couples Amino Acid Metabolism to Mitochondrial DNA Maintenance in Saccharomyces cerevisiae
Source: mBio. 2018 Feb 27;9(1):e00084-18. doi: 10.1128/mBio.00084-18 (PMC5829821; doi:10.1128/mBio.00084-18)
Supplement: TEXT S1 [file mbo001183742s1.docx]

**SI MATERIALS AND METHODS**

**Effect of serine on respiratory capacity.** A 50 µl aliquot of strain DMy20 (ρ^+^ *cha1*∆ *mmf1*∆) grown ~12 hours in YPD medium at 30˚C was used to inoculate 5 ml cultures containing SD medium with or without 5 mM serine added. Cultures were incubated at 30˚C with shaking (200 rpm) for up to 72 hours and aliquots were taken at the indicated time-points following inoculation, serially diluted to 10^5^-10^1^ in NaCl and plated (10 µl) on YPD and YPG plates. Images were acquired after ~48 hours of growth at 30˚C and are representative of two independent experiments.

**Rifampicin resistance frequency of *S. enterica* lacking *ridA*.** The *in* *vivo* mutagenicity of 2-aminoacrylate was tested in a *ridA* strain of *S. enterica* known to accumulate 2AA when grown on minimal glucose medium (17). Cultures containing minimal glucose medium (5 ml) were inoculated with a single colony of wild type *S. enterica* LT2 (DM9404) or the isogenic *ridA3::MudJ* mutant strain (DM3480). Cultures were incubated at 37˚C for 48 hours, pelleted and resuspended in NaCl solution at one-tenth the original volume and 100 µl aliquots (~10^9^ CFU) were plated on solid rich medium containing 8 g/L nutrient broth, 5 g/L sodium chloride, 15 g/L agar and 60 µg/ml rifampicin. Spontaneous rifampicin-resistant mutants were counted following 48 hours of incubation at 37˚C and the average and standard deviation from three independent experiments are reported.
