# Supplementary material for: Mmf1p Couples Amino Acid Metabolism to Mitochondrial DNA Maintenance in Saccharomyces cerevisiae
Source: mBio. 2018 Feb 27;9(1):e00084-18. doi: 10.1128/mBio.00084-18 (PMC5829821; doi:10.1128/mBio.00084-18)
Supplement: TABLE S1 [file mbo001183742st1.docx]

**TABLE S1. Plasmids and primers used in this study.**
