# Supplementary material for: Mmf1p Couples Amino Acid Metabolism to Mitochondrial DNA Maintenance in Saccharomyces cerevisiae
Source: mBio. 2018 Feb 27;9(1):e00084-18. doi: 10.1128/mBio.00084-18 (PMC5829821; doi:10.1128/mBio.00084-18)
Supplement: TABLE S2 [file mbo001183742st2.docx]

| **TABLE S2.** **Frequency of rifampicin resistant colonies in wild-type and *ridA* strains of *S. enterica*** | | |
| --- | --- | --- |
| Strain | Genotype | CFU/10^9^ cells |
| DM9404 | Wild type | 43 ± 3 |
| DM3480 | *ridA3::MudJ* | 42 ± 1 |
